# Differentiation of Human Induced Pluripotent Stem Cells Toward Implantable Chondroprogenitor Cells

**DOI:** 10.1177/19476035251351713

**Published:** 2025-07-03

**Authors:** Josefine Ekholm, Kristina Vukusic, Camilla Brantsing, Georgina Shaw, Fazal Ur Rehman Bhatti, Stina Simonsson, Anna Falk, Mary Murphy, Victoria Rotter Sopasakis, Anders Lindahl

**Affiliations:** 1 Department of Clinical Chemistry, Sahlgrenska University Hospital, Gothenburg, Sweden; 2 Department of Laboratory Medicine, Institute of Biomedicine, Sahlgrenska Academy, University of Gothenburg, Gothenburg, Sweden; 3 The Regenerative Medicine Institute, University of Galway, Galway, Ireland; 4 Department of Experimental Medical Science, Lund Stem Cell Center, Lund University, Lund, Sweden

**Keywords:** cartilage, chondrogenic differentiation, iPSC, osteoarthritis, cell therapy, ATMP

## Abstract

*Background.* Post-traumatic chondral and osteochondral lesions can be treated with autologous chondrocyte implantation (ACI), but the high cost of autologous cell expansion under strict Good Manufacturing Practice (GMP) regulations limits patient access. Stem cell–based advanced therapy medicinal products (ATMPs) offer more cost-effective alternatives, with human induced pluripotent stem cells (iPSC) showing great promise due to their expandability, low immunogenicity, commercialization potential, and fewer ethical concerns. *Aim.* To develop a protocol to direct iPSC through a mesenchymal stage into chondroprogenitors (iCHOp), resembling autologous chondroprogenitor cells used in ACI. *Methods.* The derived chondroprogenitor cells were expanded in monolayer and in 3-dimensional (3D) cultures and subsequently analyzed using transcriptomic profiling via RNA sequencing and reverse transcription quantitative polymerase chain reaction and compared with ACI chondrocytes. *Results.* Transcriptomic profiling confirmed successful differentiation, with iCHOp showing 83% similarity to ACI chondrocytes. Further 3D culture maturation led to upregulation of chondrogenesis-related genes and activation of cartilage-specific pathways. Histological analysis confirmed extracellular matrix production, including proteoglycans, collagen, and versican. Furthermore, the protocol’s reproducibility was demonstrated using 3 distinct iPSC lines, successfully expanded in both serum-containing and defined serum-free media. *Conclusion.* Our optimized approach yields iCHOp with phenotypes closely matching ACI chondrocytes, offering a solid foundation for further development and potential clinical applications in cartilage repair.

## Introduction

Post-traumatic chondral and osteochondral lesions can be effectively treated^1-3^ with autologous chondrocyte implantation (ACI).^4^ This treatment, developed in the 1990s, was among the first cell therapies designed for a broad patient population. In contrast, primary osteoarthritis (OA) is characterized by progressive breakdown of joint cartilage driven by excessive production of matrix-degrading enzymes. Due to the absence of early diagnostic tools and limited understanding of its underlying endotypes, there are currently no disease-modifying treatments for primary OA.

In large cohort studies of patients undergoing arthroscopies, cartilage lesions were identified in 60% of cases, with 7% to 9% of individuals aged 40 to 50 requiring regenerative repair.^5^ This underscores the urgent need for effective treatment. Traumatic injuries to the joint predominantly afflicts younger patients and, if left untreated, can lead to post traumatic OA and joint destruction in the affected knee. The NICE (National Institute for Health and Care Excellence) report from 2017 (Technology appraisal guidance TA477) emphasizes the importance of regenerative treatments for traumatic local cartilage defects.^6^

Long-term follow-up studies of ACI treatments have shown promising outcomes, with 90% of treated patients maintaining normal knee function even 10 to 20 years post-treatment.^1,7,8^ However, ACI’s high cost limits its accessibility, highlighting the need for scalable and affordable solutions. Developing cost-effective, off-the-shelf products could significantly increase access to treatment and reduce the burden of severe localized traumatic injuries. Combining cells with biomaterials^9,10^ or 3D-printed scaffolds presents an exciting avenue for arthroscopic treatments, reducing costs and enabling advanced cell-based therapies as off-the-shelf advanced therapy medicinal products (ATMP). Previously, our research demonstrated the derivation of chondrocytes from embryonic stem cells when cocultured with irradiated human chondrocytes from ACI^11^ and from mRNA-induced iPSC derived from human chondrocytes.^12^ These studies successfully showed cartilage development in 3D cultures.

Over the past decade, several protocols have emerged for chondrocyte differentiation from iPSC cells, primarily via 3 pathways: embryoid body formation, via mesenchymal stem cells (MSCs),^13^ mesodermal differentiation,^14^ and neural crest pathways.^15^ While effective and reproducible, these protocols often involve complex culture techniques and costly additives, making large-scale, automated production challenging. Our aim was to simplify the chondrocyte differentiation protocol, reducing it to essential factors while eliminating xeno-additives, such as fetal bovine serum, to create a scalable, Good Manufacturing Practice (GMP)-compliant cell production system.

Our optimized protocol directs the differentiation of iPSC into human mesenchymal-like cells, integrating neural crest and mesodermal stimulation using an SMAD inhibitor and FGF10. The transcriptomic profile of the induced chondroprogenitors (iCHOp) was analyzed using RNA sequencing and reverse transcription quantitative polymerase chain reaction (RT-qPCR), assessing relevant signaling pathways and mesenchymal and chondrogenic gene expression. The data were compared with monolayer-expanded chondrocytes isolated from adult cartilage during ACI procedures. Synthesis of extracellular matrix (ECM) components was further evaluated by histological analysis and immunofluorescence. In addition, reproducibility and GMP compliance of the protocol were tested by generating iCHOp from 3 distinct iPSC lines grown in xeno-free medium without serum.

## Materials and Methods

The research was performed in accordance with relevant guidelines and regulations.

### Human iPSC Culture

A human iPSC line, A2B, derived by reprogramming human articular chondrocytes,^12^ was used as the primary experimental cell line during development and optimization of the chondrogenic differentiation protocol. To evaluate the reproducibility of the chondrogenic differentiation protocol, the process was applied to 2 additional iPSC lines. The first, CTRL-10-I, was a GMP-derived cell line, developed at the Vecura facility at Karolinska Hospital and at Karolinska Institute, Stockholm, Sweden^16^ and kindly provided by Professor Anna Falk. The second was ChiPSC22, a commercially available cell line purchased from Takara Bio Europe AB, Gothenburg, Sweden, included to further validate the protocol.

All 3 cell lines were expanded on Corning Primaria cell culture using multiwell plates and Corning Primaria tissue culture flasks (Fisher Scientific, Waltham, MA), coated with DEF-CS 500 COAT-1 (Takara Bio Europe AB, Gothenburg, Sweden), according to the manufacturer’s protocol. The cells were cultured in Cellartis DEF-CS 500 Basal Medium with additives (Takara Bio Europe AB, Gothenburg, Sweden) with a seeding density of 25,000 cells/cm^2^. The medium was changed once a day. Passage of cells was done at 80% to 90% confluence using TrypLE Select Enzyme (Gibco/Thermo Fisher Scientific, Waltham, MA).

### Human Adult Chondrocyte Cell Culture

Research ethics approval (S 040-01) was granted by the Research Ethics Board at the Sahlgrenska Academy, University of Gothenburg, Sweden, and all study procedures followed the ethical guidelines of the declaration of Helsinki. Human adult articular chondrocytes grown in monolayer or 3D were used as reference cells and donated by the Cell and Tissue Laboratory, Sahlgrenska University Hospital, Gothenburg, Sweden. The chondrocytes were harvested and isolated from biopsies from knee joints of 3 different patients (18-25 years of age) undergoing ACI treatment,^4^ anonymized and donated for research after ACI. A signed informed consent was obtained from each patient.

Briefly, chondrocytes were expanded in monolayer in DMEM/F12 medium (Invitrogen, Waltham, MA) supplemented with 10% human serum (pooled from blood donors), l-ascorbic acid (25 µg/ml Apoteket, Umeå, Sweden), 1% penicillin/streptomycin (PAA Laboratories, Toronto, Canada), and 2 mM l-glutamin (Invitrogen, Waltham, MA) and used in passages 2 to 4.

### Chondrogenic Differentiation (Step 1)—14 Days in Monolayer Culture

**Figure 1.**
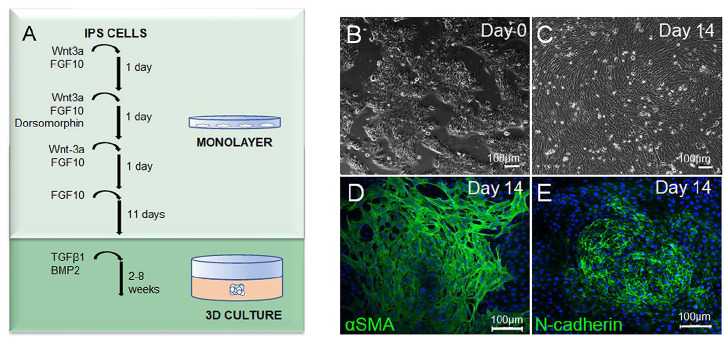
**(A)** Simplified overview of the differentiation protocol of iPSC into human induced chondrocyte progenitors (iCHOp). **(B-E)** Morphology of the monolayer of the iPS-A2B cell line. Images show cells at the start of differentiation **(B)** and after 14 days of differentiation **(C)**. Immunocytochemistry on fixed monolayer at day 14 of differentiation, showing expression of mesodermal markers alpha smooth muscle actin (αSMA) **(D)** and N-cadherin **(E)** in green. Nuclei were stained blue with DAPI.

When the cells reached confluence, the medium was changed to DMEM, low glucose, GlutaMAXmedium, supplemented with 2% human serum, reagents and growth factors, specified in **Table 1.** To stimulate differentiation of the cells to iCHOp, FGF10, Wnt3a, and dorsomorphin were added according to **Table 1** and **Figure 1A**. The cells had a complete medium change once a day (2 ml/well) during the initial 14 days of differentiation in monolayer.

**Table 1.** Cell Culture Media for the Chondrogenic Differentiation Protocol.

Differentiation Medium Monolayer	Manufacturer	Catalog Number	Working Conc
DMEM, low glucose, GlutaMAX	Gibco	21885-108	1X
Sodium pyruvate	Sigma Aldrich	P5280	1 mM
Penicillin/streptomycin	Hyclone	SV30010	1%
human serum	Pool from donors	None	2%
l-Ascorbic acid 2-phosphate sesquimagnesium salt hydrate	Sigma Aldrich	A8960-5G	80 µM
**Day 0**			
FGF10	R&D Systems	345-FG-025	4 µM
Wnt 3A	R&D Systems	5036-WN	4 µM
**Day 1**			
FGF10	R&D Systems	345-FG	10 ng/ml
Wnt 3A	R&D Systems	5036-WN-010	10 ng/ml
Dorsomorphin dihydrochloride	Bio-Techne	P5499	4 µM
**Day 2**			
FGF10	R&D Systems	345-FG	10 ng/ml
Wnt 3A	R&D Systems	5036-WN-010	10 ng/ml
**Day 3-14**			
FGF10	R&D Systems	345-FG	10 ng/ml
Differentiation medium for 3D culture	Manufacturer	Catalog number	Working conc.
Dulbecco’s Modified Eagle’s Medium—high glucose	Gibco	D6171	1x
Sodium pyruvate	Sigma Aldrich	P5280	1 mM
l-glutamine	Gibco	25030-024	2mM
Penicillin/streptomycin	HyClone	SV30010	1%
Insulin-Transferrin-Selenium	Gibco	41400045	1X
Human serum albumin	Europa Bioproducts Ltd	EQHSA62	1 mg/ml
Linoleic acid	Sigma Aldrich	L1012	5 µg/mL
l-Ascorbic acid 2-phosphate sesquimagnesium salt hydrate	Sigma Aldrich	A4403	80 µM
Dexamethasone	Sigma Aldrich	31375	100 nM
Recombinant Human BMP-2	R&D Systems	355-BM-050	100 ng/ml
Recombinant Human TGF-beta 1	R&D Systems	240-B-010/CF	10 ng/ml

**Table 1** summarizes the contents of the different cell culture media and reagents used during chondrogenic differentiation of iPSC with the described protocol.

The proliferative capacity of iCHOp-A2B, iCHOp-CTRL-10-I, and iCHOp-ChiPSC22 was evaluated after the initial 14 days of chondrogenic differentiation in monolayer culture. The cells were expanded in either a serum-containing medium or the proprietary xeno- and serum-free medium, PurStem 2 (alpha MEM, ascorbic acid-2 phosphate, dexamethasone, low-density lipoprotein, human serum albumin, FGF2, and TGFβ1),^17^ to assess its ability to support cell expansion under GMP-compliant conditions, demonstrating its potential suitability for future clinical-grade cell production.

### Chondrogenic Differentiation (Step 2)—3D Culture

To stimulate the iCHOp obtained in Step 1 into more mature chondrocytes, iCHOp were cultured in 3D as free floating pellets,^18^ with a median range of 14 days, in the presence of factors with beneficial effects on growth and chondrogenic differentiation (**Table 1**). Human adult articular chondrocytes served as reference cells. At day 14 in monolayer culture, the cells were detached with 0.05% Trypsin-EDTA (Thermo Fisher Scientific, Waltham, MA), washed with phosphate-buffered saline (PBS), counted, pelleted by centrifugation (500*g* for 5 min) and seeded with a cell density of 200,000 cells per well on Corning Costar Ultra-Low Attachment 96-well plates (Merck, Rahway, NJ), allowing self-assembly into 3D spheroids. The medium (**Table 1**) was changed 3 times/week (100 μl/well). The 3D cultures were harvested at different time points for RNA isolation for RT-qPCR and RNA sequencing analysis and for histology/immunohistochemistry.

### Immunocytochemistry

Cells in monolayer culture were washed with PBS and fixed with Histofix (Histolab Products AB, Sweden) for 20 min. Permeabilization was performed with 0.1% Triton X-100 (Sigma-Aldrich, Saint Louis, MO) in PBS for 10 min and blocking with 2% bovine serum albumin (Sigma-Aldrich, Saint Louis, MO), followed by 0.75% glycine and 0.1% Triton X-100 (Sigma-Aldrich, Saint Louis, MO) in PBS for 15 min. Primary antibodies for α-SMA, N-cadherin, Nanog, and OCT4 (Supplemental **Table 1**) were diluted in blocking solution and incubated with the sections in a humidified chamber at 4 °C overnight. The cells were then washed with PBS and incubated for 2 h at room temperature (RT) with Alexa Fluor 488 secondary antibodies (Invitrogen). Finally, cells were washed with PBS and mounted with ProLong Gold Antifade Reagent with DAPI (Life Technologies, Carlsbad, CA).

### RNA Isolation

Total RNA was extracted from adult chondrocytes, iPSC and iCHOp cultured in monolayer or 3D for 2 to 4 weeks. The 3D spheroids were disrupted by sonication using a TissueLyser (Qiagen, Hilden, Germany). To shear genomic DNA a QIA shredder column (Qiagen, Hilden, Germany) was used prior to RNA cleanup. Total RNA was extracted using RNeasy Mini Kit (Qiagen, Hilden, Germany) according to the manufacturer’s protocol.

### mRNA Sequencing

RNA integrity was assessed using capillary electrophoresis (Fragment Analyzer, Advanced Analytical). Libraries were prepared using the QuantSeq 3‘mRNA-Seq Library Kit (FWD) for Illumina (Lexogen GmbH, Vienna, Austria). The libraries were sequenced using the NextSeq500 platform (Illumina) at the TATAA Biocenter, Gothenburg, Sweden.

### Bioinformatic Analyses Following RNA Sequencing

The raw data were aligned with the human GRCh38.90 reference library from the Ensembl genome browser (https://www.ensembl.org/Homo_sapiens/Info/Index), and the resulting BAM files were used for bioinformatics analysis. Transcripts with counts ≥5 in at least 15% of the samples were included in the bioinformatics analysis. Statistical analysis, principal component analysis (PCA), and gene set enrichment analysis (GSEA)^19^ of the gene data were performed with Qlucore Omics Explorer 3.6 (Qlucore AB, Lund, Sweden). The Molecular Signatures Database (https://www.gsea-msigdb.org/gsea/msigdb/index.jsp) was used to obtain gene sets for GSEA. Levels of significance for differences between group means were determined with *t* test or 2-way analysis of variance. A false discovery rate–adjusted *P*-value (=*q*-value) < 0.05 was considered significant.

### RT-qPCR

The cDNA was prepared from total RNA using High-Capacity cDNA Reverse Transcription Kit (Applied Biosystems, Waltham, MA). RT-qPCR analysis was performed with the ABI Prism sequence detection system (Applied Biosystems, Waltham, MA). Human TaqMan Gene Expression Assays (Applied Biosystems, Waltham, MA) are summarized in Supplemental **Table 2**. Samples were analyzed using 2 technical replicates. The relative comparative method was used to analyze the RT-qPCR data (Sequence Detector User Bulletin 2; Applied Biosystems, Waltham, MA) and the relative quantification values were calculated using CYPA as the reference gene and an in-house calibrator sample. Gene expression data are presented in relative units.

### Histology

The 3D spheroids were fixed in Histofix (Histolab Products AB), dehydrated, and embedded in paraffin. Six-micrometer sections were cut and placed onto glass slides (Superfrost Plus, Menzel-Gläser), deparaffinized and stained with Alcian blue van Gieson. A Nikon Eclipse 90i microscope (Nikon, Minato, Japan) and NIS Elements AR software version 3.22.11 were used for visualization.

### Immunohistochemistry

Deparaffinized and rehydrated sections were washed in PBS followed by an antigen retrieval step with preheated (approx. 90 °C) 0.1M citrate buffer, pH 6.0, for 20 min. The sections were kept at 70 °C for 30 min and at RT for an additional 30 min. Digestion with hyaluronidase (8000 U/ml) (Sigma-Aldrich, Saint Louis, MO) for 1 h at 37 °C was performed followed by incubation with chondroitinase ABC (0.05 U/ml) (Sigma-Aldrich, Saint Louis, MO) for 1 h at 37 °C (chondroitinase incubation was not performed for Collagen III antibody analysis). Sections were then rinsed in PBS and blocked with 10% goat serum (Invitrogen, Waltham, MA) in PBS for 1 h. Primary antibodies were diluted in blocking solution according to Supplemental **Table 1** and incubated in a humidified chamber at 4 °C overnight.

The sections were washed with PBS and incubated for 2 h at RT with secondary Alexa Fluor 546 antibodies (Invitrogen, Waltham, MA). Finally, sections were washed with PBS and mounted with ProLong Gold Antifade Reagent with DAPI (Life Technologies, Carlsbad, CA).

## Results

The A2B iPSC line, derived from human chondrocytes, has previously been characterized and shown to express pluripotency markers and the ability to differentiate into tissues representing the 3 different germ layers.^12^ In the current study, as a first control step, the pluripotency of the iPSC-A2B line^12^ was confirmed through the expression of the stem cell markers NANOG and Oct4 using immunocytochemistry on the confluent monolayers of iPSC-A2B (Supplemental **Fig. 1**).

### Dorsomorphin, Wnt3a, and FGF10 are Crucial for Chondrogenic Differentiation of iPSC

During optimization we observed that 100% confluence was required at the start of differentiation for effective chondrogenic induction. The iPSC underwent a 2-step chondrogenic differentiation; an initial differentiation in monolayer for 14 days, followed by cultivation in 3D for up to 8 weeks (**Fig. 1A**). A stepwise optimization of added factors and their concentrations was performed and their impact on the chondrogenic differentiation evaluated (data not shown). Dorsomorphin, Wnt3a, and FGF10 were crucial stimulators for the differentiation of iPSC. From here on, we refer to differentiated iPSC as iCHOp. iCHOp morphology and expression of the mesodermal marker αSMA and the epithelial-to-mesenchymal transition marker N-cadherin after 14 days of differentiation in monolayer are shown in **Figure 1B-E**. The thymic fibroblast marker TE7 or hematopoietic marker CD45 was not detected (data not shown). We refer to iCHOp grown in differentiation media in monolayer during the initial 14 days as *iCHOp mono* and the subsequently cultured spheroids as *iCHOp 3D*.

### iCHOp Display Gene Expression Patterns Concordant with Chondrogenic Differentiation

**Figure 2.**
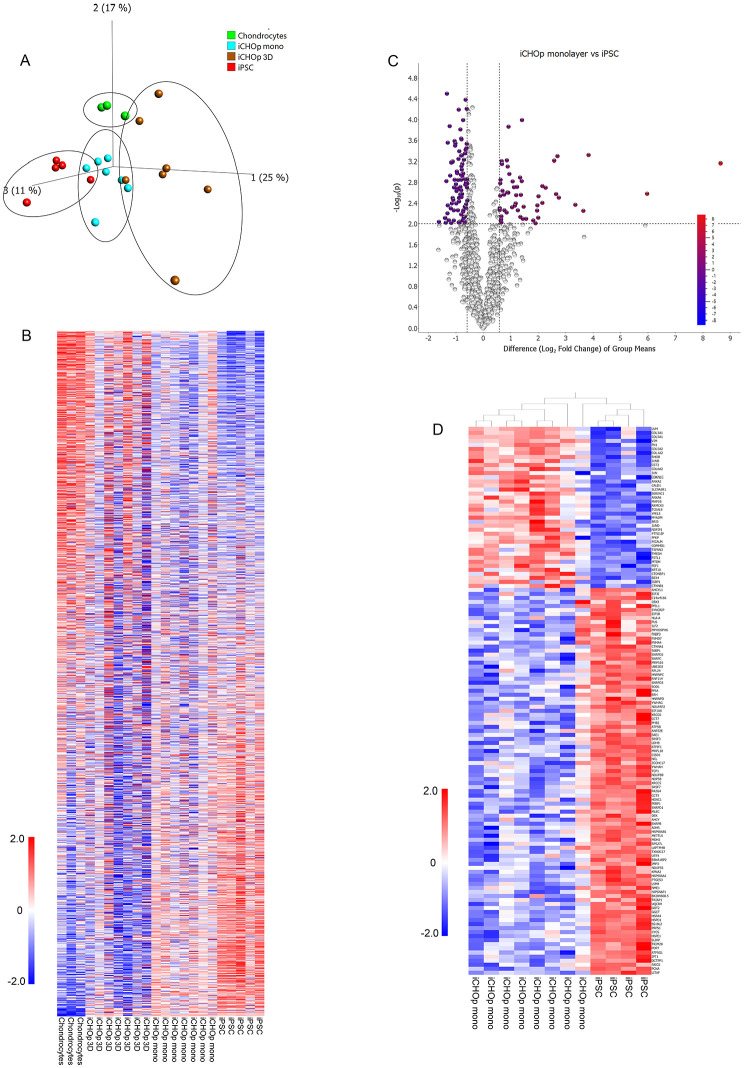
Differential gene expression by RNA sequencing between undifferentiated iPSC in monolayer, iCHOp in monolayer (iCHOp mono), iCHOp after maturation in 3D, and adult primary human chondrocytes in monolayer. **(A)** PCA score plot (first 3 components) of the total number of genes detected, showing a separation between the groups, **(B)** Heat map of all genes detected for all groups, **(C)** Volcano plot displaying the difference in gene expression (log2 fold change, *q*-value < 0.05) between iPSC and iCHOp mono, **(D)** Heatmap of genes differing significantly between iPSC and iCHOp mono (log2 fold change, *q*-value < 0.05). *n* = 5 for iPSC, *n* = 7 for iCHOp mono, *n* = 7 for iCHOp 3D, and *n* = 3 for chondrocytes.

To assess the ability of our protocol to drive iPSC-A2B toward the chondrogenic lineage, and to obtain a comprehensive insight of gene expression patterns during differentiation, we performed RNA sequencing. We compared gene expressions of iPSC, iCHOp mono, iCHOp 3D at different stages (with a median range of 14 days) as well as adult human chondrocytes grown in monolayer from 3 different patients. Using PCA, an unbiased multivariate classification model, we observed a separation between all groups with regard to gene expression (**Fig. 2A**). Of the 1,523 transcripts detected, 457 transcripts differed between adult chondrocytes and iPSC (**Fig. 2B**). In total, 136 genes showed significantly altered expression in the iCHOp mono group compared with iPSC (**Fig. 2C** **-D**). GSEA revealed pathways related to ECM organization, collagen and proteoglycan biosynthesis among the top-scored pathways differing between iCHOp mono and iPSC (**Table 2**), indicating a significant transformation of iCHOp in monolayer toward differentiated chondrocytes.

**Table 2.** Gene Set Enrichment Analysis of iCHO Mono versus iPSC.

Reactome Pathway	NES	*P*	*q*
COLLAGEN DEGRADATION	1.71	0.004	0.16
ECM PROTEOGLYCANS	1.70	0.004	0.15
DEGRADATION OF THE EXTRACELLULAR MATRIX	1.69	0.0041	0.16
NON INTEGRIN MEMBRANE ECM INTERACTIONS	1.68	0	0.14
HS GAG DEGRADATION	1.66	0	0.13
EXTRACELLULAR MATRIX ORGANIZATION	1.64	0.004	0.13
CROSSLINKING OF COLLAGEN FIBRILS	1.63	0	0.11
MOLECULES ASSOCIATED WITH ELASTIC FIBERS	1.63	0.0021	0.11
ELASTIC FIBER FORMATION	1.63	0.0021	0.12
MET PROMOTES CELL MOTILITY	1.61	0	0.11
O GLYCOSYLATION OF TSR DOMAIN CONTAINING PROTEINS	1.61	0.0040	0.12
COLLAGEN CHAIN TRIMERIZATION	1.59	0.0060	0.13
HEPARAN SULFATE HEPARIN HS GAG METABOLISM	1.57	0.0042	0.13
COLLAGEN BIOSYNTHESIS AND MODIFYING ENZYMES	1.56	0.010	0.13
COLLAGEN FORMATION	1.55	0.014	0.14
ASSEMBLY OF COLLAGEN FIBRILS AND OTHER MULTIMERIC STRUCTURES	1.55	0.015	0.15
ACTIVATION OF MATRIX METALLOPROTEINASES	1.54	0.047	0.15
SYNDECAN INTERACTIONS	1.52	0.0059	0.16
DISEASES OF GLYCOSYLATION	1.52	0.012	0.17
DISEASES ASSOCIATED WITH GLYCOSAMINOGLYCAN METABOLISM	1.51	0.015	0.17
LAMININ INTERACTIONS	1.48	0.033	0.19
SIGNALING BY PDGF	1.49	0.014	0.19
GLYCOSAMINOGLYCAN METABOLISM	1.46	0.017	0.21
O LINKEDGLYCOSYLATION	1.44	0.038	0.24
RUNX2 REGULATES OSTEOBLAST DIFFERENTIATION	1.44	0.044	0.24

Pathways differing between iCHO in monolayer and undifferentiated iPSC.

NES = normalized enrichment score; *q*-value = adjusted *P*-value.

iCHOp mono shared 83% similarity in gene expression with adult chondrocytes grown in monolayer. Among the genes that differed between these 1 groups were genes involved in cholesterol biosynthesis, cell proliferation, migration and differentiation as assessed by GSEA (**Table 3** and Supplemental **Table 3**), although the GSEA *q*-values did not reach the desired cut off, suggesting that no pathways were strongly different between these 2 groups. Many of the differentially expressed genes that were upregulated in iCHOp mono compared with adult chondrocytes are involved in neuronal development (Supplemental **Table 3**), which may suggest a neuroectodermal origin of these cells.

**Table 3.** Gene Set Enrichment Analysis of iCHO Mono versus Chondrocytes.

Reactome Pathway	NES	*P*	*q*
REGULATION OF MECP2 EXPRESSION AND ACTIVITY	1.39	0.02	0.67
REGULATION OF CHOLESTEROL BIOSYNTHESIS BY SREBP SREBF	1.39	0.03	0.68
SYNTHESIS OF PC	1.41	0.02	0.68
SIRT1 NEGATIVELY REGULATES RRNA EXPRESSION	1.41	0.09	0.68
NRAGE SIGNALS DEATH THROUGH JNK	1.39	0.02	0.69
CHOLESTEROL BIOSYNTHESIS	1.41	0.09	0.69
SUMOYLATION OF CHROMATIN ORGANIZATION PROTEINS	1.40	0.06	0.69
SIGNALING BY NODAL	1.39	0.05	0.69
KINESINS	1.38	0.06	0.70
SIGNALING BY NOTCH1	1.39	0.03	0.70
SIGNALING BY NOTCH1 IN CANCER	1.35	0.05	0.70
EPH EPHRIN MEDIATED REPULSION OF CELLS	1.40	0.04	0.70
CELL JUNCTION ORGANIZATION	1.35	0.05	0.70
HS GAG BIOSYNTHESIS	1.35	0.06	0.70
CRMPS IN SEMA3A SIGNALING	1.38	0.05	0.70
HDMS DEMETHYLATE HISTONES	1.41	0.05	0.70
LONG TERM POTENTIATION	1.35	0.08	0.70
ACTIVATED NOTCH1 TRANSMITS SIGNAL TO THE NUCLEUS	1.35	0.08	0.70
SYNAPTIC ADHESION LIKE MOLECULES	1.35	0.07	0.71
TRANSPORT OF VITAMINS NUCLEOSIDES AND RELATED MOLECULES	1.36	0.02	0.71
FOXO MEDIATED TRANSCRIPTION OF CELL DEATH GENES	1.34	0.08	0.71
OXIDATIVE STRESS INDUCED SENESCENCE	1.40	0.03	0.71
RHO GTPASES ACTIVATE PKNS	1.36	0.1	0.71
PHOSPHORYLATION SITE MUTANTS OF CTNNB1 ARE NOT TARGETED TO THE PROTEASOME BY THE DESTRUCTION COMPLEX	1.38	0.05	0.71

Pathways differing between iCHO in monolayer and chondrocytes in monolayer.

NES = normalized enrichment score; q-value = adjusted *P*-value.

### Chondrogenic Maturity of iCHOp Increased in 3D Cultures

To assess the chondrogenic maturity during differentiation in monolayer and 3D, we compared the full transcriptome of iCHOp mono, iCHOp 3D and adult chondrocytes. PCA revealed distinct clustering of the different cell categories (**Fig. 2A**), indicating variations in chondrogenic maturity based on gene expression profiles. To search for genes directly involved in cartilage development, we used the Gene Ontology (GO) resource. In search for enrichment of genes in iCHOp associated with a particular cellular component, regulatory pathway, biological function, and so on, we found genes associated with cartilage development and embryonic differentiation of progenitor cells into the chondrogenic lineage (**Table 4**).

**Table 4.** Gene Ontology Enrichment Analysis.

Annotation Data Set	Pathway	Fold Enrichment	*P*-value
GO Cellular component	Collagen trimer	2.45	1.9E-03
	Collagen-containing extracellular matrix	2.20	8.2E-07
GO Biological process	Regulation of Wnt signaling pathway, planar cell polarity pathway	5.61	8.9E-04
	Notochord development	4.73	2.0E-03
	Embryonic forelimb morphogenesis	3.73	1.9E-03
	Embryonic skeletal system morphogenesis	3.51	4.2E-07
	Mesenchymal cell development	3.21	1.7E-05
	Positive regulation of epithelial to mesenchymal transition	3.14	1.2E-03
	Mesenchymal development	3.04	5.6E-11
	Regulation of BMP signaling pathway	2.90	6.2E-07
	Embryonic digit morphogenesis	2.88	1.6E-03
	BMP signaling pathway	2.87	3.3E-04
	Regulation of cartilage development	2.85	5.2E-04
	Extracellular matrix organization	2.28	1.4E-06
	Cellular response to TGFβa stimulus	2.12	1.9E-03
	Cartilage development	2.10	1.2E-03

GO enrichment analysis (over-representation test) using the analysis tool from the Panther classification system (Fisher’s exact, false discovery rate *P* < 0.05).

**Figure 3.**
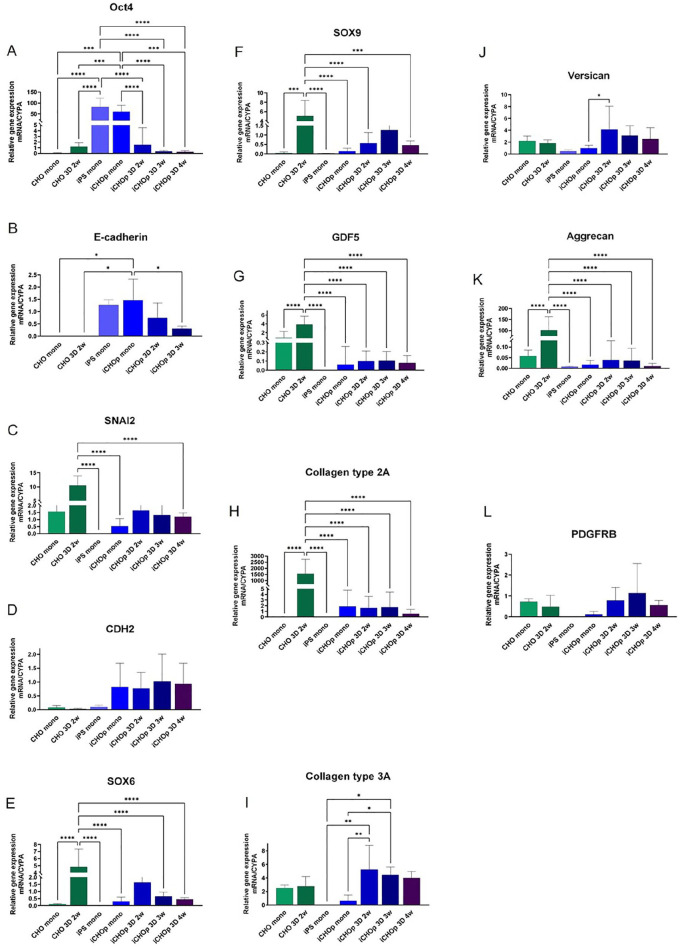
Expression of genes involved in iCHOp differentiation toward cartilage. mRNA expression by qPCR of genes involved in Epithelial-mesenchymal transition and chondrogenesis (A-L), normalized to CYPA mRNA. CHO mono = adult human chondrocytes in monolayer, CHO 3D = chondrocytes grown in 3D for 2 weeks, iPS mono = undifferentiated iPS cells in monolayer, iCHOp = iCHOp grown in monolayer, iCHOp 3D = iCHOp grown in 3D for 2 to 4 weeks. Error bars indicate standard deviation, *n* = 3 to 4 for chondrocytes, *n* = 3 to 5 for iPSCs, *n* = 3 to 18 for iCHOp. *P < 0.05, **P < 0.001, ***P < 0.0005, ****P < .0001.

To confirm the RNA-seq data of chondrogenic development, we quantified chosen markers of pluripotency and differentiation using RT-qPCR (**Fig. 3**). The results showed a downregulation of Oct4 and E-cadherin (**Fig. 3A-B**) and a tendency toward upregulation of SNAI2 and N-cadherin (CDH2) (**Fig. 3C-D**) during differentiation in 3D, suggesting that the iPSC had transformed into mesodermal cells. The cells continued to differentiate toward chondrocytes as shown by expression of SOX 6 and 9 genes and GDF5 (**Fig. 3E-G**) early in 3D differentiation followed by collagen type 2 and 3 (**Fig. 3H-I**). Of the proteoglycans, versican levels were higher in iCHOp 3D compared with aggrecan (**Fig. 3J-K**), suggesting that the iCHOp 3D displayed a more fetal, rather than adult, phenotype. The receptor for chemoattractant, PDGFB, showed a tendency of higher expression during 3D differentiation (**Fig. 3L**).

**Figure 4.**
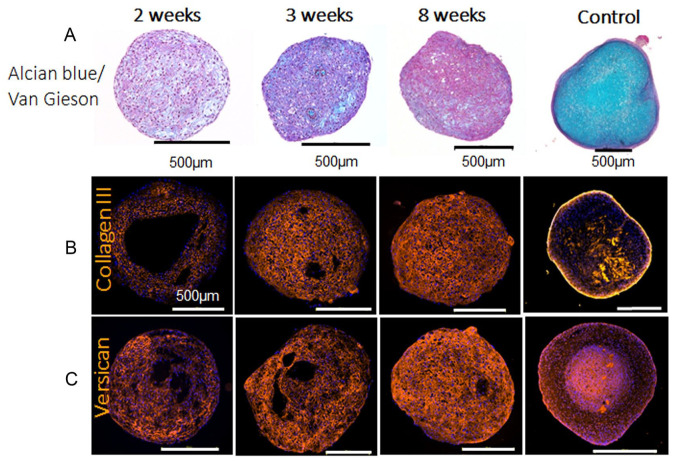
Histology and immunohistochemistry of iCHOp grown in 3D. Cell spheroids after 2, 3, and 8 weeks in 3D culture. **(A)** Extracellular components in cultured iCHOp visualized by Alcian blue van Gieson staining where proteoglycans appear blue and collagens purple. **(B-C)** 3D cultures of iCHOp were stained with antibodies for collagen type III **(B)** and versican **(C)**. The right column shows a human chondrocyte spheroid as a positive control sample.

Histology staining with Alcian blue van Gieson verified increasing proteoglycan and collagen production over time in iCHOp 3D cultures (**Fig. 4A**). Furthermore, immunohistochemistry results verified expression of collagen type 3 (**Fig. 4B**) and versican (**Fig. 4C**) during differentiation in 3D. Aggrecan expression, however, remained low during the 3D differentiation (data not shown).

Taken together, RT-qPCR analysis confirmed differentiation toward cartilage using our protocol. Furthermore, key ECM components were expressed in increasing amounts during differentiation of iCHOp in 3D, although with a phenotype resembling that of fetal nature, with high expression of versican and low expression of aggrecan.

### iCHOp Grown in Monolayer Proliferate in Serum-Free Medium Following Differentiation

**Figure 5.**
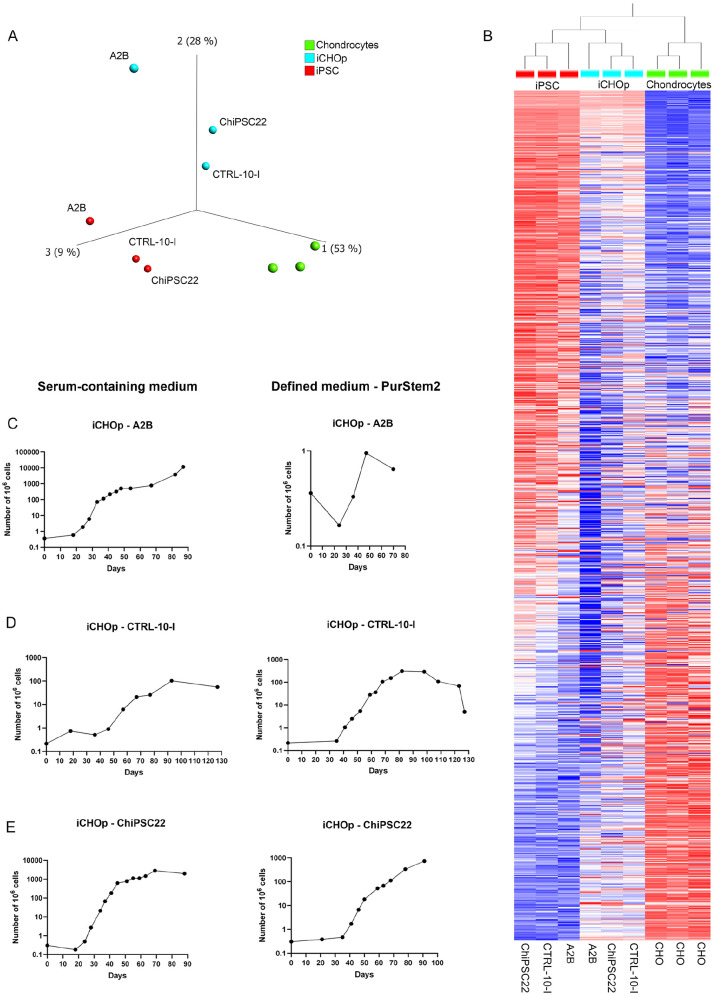
Reproducibility of the differentiation protocol and ability of iCHOp to proliferate in serum-free medium. **(A)** PCA (first 3 components) of gene expression in iPSC and iCHOp for the 3 cell lines following RNA sequencing, **(B)** Gene heatmap for iPSC and iCHOp of all 3 cell lines. **(C-E)** Cumulative cell growth of 3 different iPS cell lines in serum-free medium (PurStem 2) compared with serum-containing medium.

To test the reproducibility of our differentiation protocol, we performed RNA sequencing of 3 different iPSC lines and the resulting iCHOp following differentiation for 14 days in monolayer and compared gene expression by PCA. In addition to our A2B cell line derived from human chondrocytes described above, we included a GMP-derived iPSC line, CTRL-10-I,^20^ and commercially available ChiPSC22, derived from human skin fibroblasts. We confirmed a distinct separation between iPSC, iCHOp mono, and human adult chondrocytes (**Fig. 5A**). The different cell lines, however, clustered together within the iPSC and iCHOp groups, indicating that differentiation maturity was similar among the different cell lines with regard to gene expression (**Fig. 5A-B**).

Important goals of this study were to adapt the production of cells for scale up in an automatized GMP platform and to expand cells in a defined, xeno-free medium that is GMP-compliant and suitable for future cell therapy. We therefore expanded iCHOp derived from the 3 different iPSC lines in both serum-containing medium and a defined medium without serum (PurStem 2) following differentiation for 14 days with our differentiation protocol. Both the CTRL-10-I-iCHOp and the ChiPSC22-iCHOp showed proliferative capacity in PurStem 2, whereas A2B-iCHOp did not (**Fig. 5C-E**). All 3 cell lines had the highest number of cell doublings at passage 4, irrespectively of cell culture medium.

**Figure 6.**
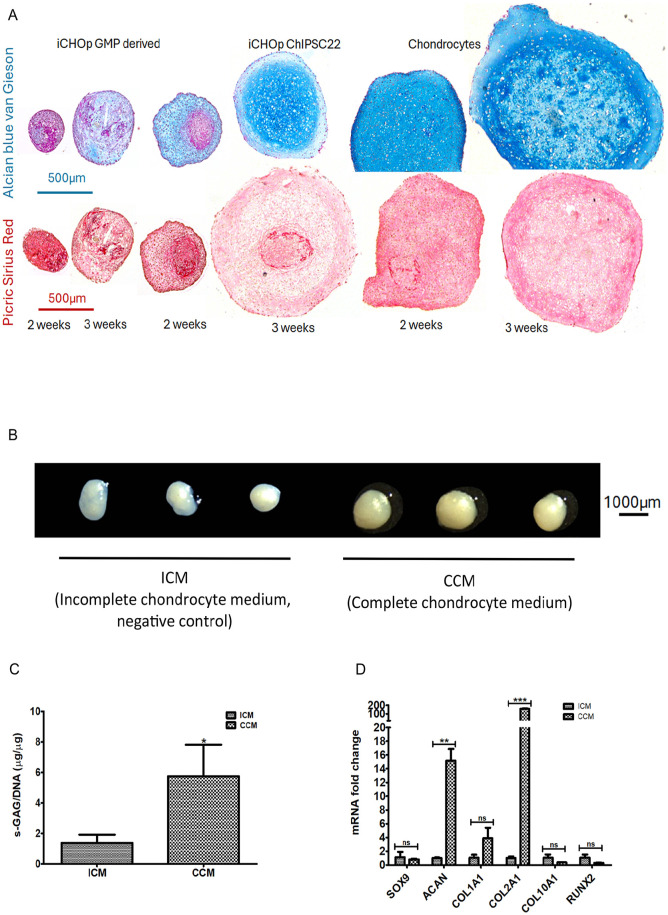
Chondrogenic maturation of iCHOp after expansion in a xenofree medium. **(A)**, Histological analysis of cell spheroids following 2 and 3 weeks in 3D culture was performed. Extracellular components are visualized by Alcian blue van Gieson staining (upper panel), where proteoglycans appear blue and collagens purple, and Picric Sirius staining (lower panel), which stains collagen red. The samples analyzed included iCHOp derived from ChiPSC22, iCHOp derived from GMP-grade CTRL-10-I cells, and adult chondrocytes, used as positive controls. The reproducibility of the protocol using PurStem 2 was assessed by replicating the 3D experiment with the ChiPSC22 cell line in a different laboratory. This independent validation confirmed pellet formation **(B)** and the production of ECM components **(C-D)**. ICM = incomplete chondrocyte medium, which lacks TGFβ1, used as a negative control. CCM = complete chondrocyte medium. *P < 0.05, **P < 0.01, ***P < 0.001 (Student’s *t* test).

Following expansion in PurStem 2 medium, the iCHOp mono were cultured in differentiation medium for 3D culture (**Table 1**) and compared with adult chondrocytes. Histological staining with Alcian blue van Gieson and Picric Sirius red showed further maturation of iCHOp with proteoglycan and collagen production after 2 and 3 weeks in pellet cultures (**Fig. 6A**). All showed an increase in proteoglycan production between 2 and 3 weeks and a decrease in the intensity of collagen staining. The reproducibility of the protocol using PurStem 2 was further evaluated by conducting the 3D experiment with the ChiPSC22 cell line in a different laboratory. This independent replication confirmed pellet formation (**Fig. 6B**) and the production of ECM components (**Fig. 6C-D**).

## Discussion

ACI is an ATMP, developed to address the medical need for treating traumatic chondral or osteochondral lesions.^4^ While ACI has demonstrated effectiveness and successful long-term outcomes in managing traumatic cartilage lesions,^2,3^ the treatment process is labor-intensive, requiring meticulous laboratory work. The high cost per patient arises from the need for rigorous quality control of each autologous cell batch under strict GMP regulations.

Primary OA (an organ disease) can be seen as due to natural aging and wear and tear of the joint over time. No specific trigger or underlying disease is identified but it is thought to involve genetic predisposition. Primary OA is thus a systemic disease associated with increased mortality and comorbidities such as heart disease and dementia. Secondary OA (also an organ disease) results from an identifiable factor or underlying condition that damages the joint, such as injury or trauma (like osteochondral fractures, in conjunction with meniscus lesions or ligament tear). Obesity, inflammatory diseases (e.g., rheumatoid arthritis), metabolic disorders (e.g., hemochromatosis, diabetes), congenital joint abnormalities (e.g., hip dysplasia). It is important to emphasize that ACI is not indicated neither for primary nor for secondary OA. ACI is indicated for local cartilage traumatic defects but a successful repair with ACI may hinder or slow down a progression of a local cartilage defect into a secondary OA. Future cost-effective ATMP solutions for traumatic cartilage injuries—and potentially secondary OA if combined with systemic anti-inflammatory drugs—require cells suitable for large-scale culture in bioreactors or automatized cell factories. Such products should ideally be off-the-shelf and based on chondrocytes incorporated into scaffolds that facilitate arthroscopic implantation. Key requirements for these cells include the ability to integrate with native cartilage, avoid immune responses or tumorigenesis, as well as provide mechanical strength and elasticity to withstand wear and tear.

Adult chondrocytes in articular cartilage have limited proliferative capacity and are unable to regenerate neocartilage following injury. However, recent research has highlighted a limited repair capacity from resident stem cells in the superficial layer of cartilage. The understanding of these endogenous stem/progenitor cells (ESPCs) has advanced significantly in recent years (see review).^21^ Studies have demonstrated the involvement of joint resident ESPCs in the cartilage repair processes.^22^ Whether these cells share characteristics with expanded chondrocytes used in ACI remains to be determined. New cell sources, potentially in combination with extracellular vesicles, are crucial for broader OA treatment strategies. In addition, improved early diagnostic tools, such as companion diagnostics, are essential.^23^ Recently, our group identified novel early biomarkers for OA in horses, which have direct human relevance due to sequence homology.^24^ These findings could pave the way for earlier detection and intervention in OA.

MSCs, isolated from bone marrow or adipose tissue have been extensively studied for their potential in treating OA.^25,26^ The injection of MSCs into the joint have been based on a hypothesis that the cells should secrete growth factors and enzymes that might reduce the OA progress. Subsequently, not to be cells injected directly in local defects and produce repair tissue. While clinical trials have demonstrated their ability to reduce inflammation in many patients, MSCs have not progressed to a disease-modifying treatment due to significant variability between cell batches.^27^ To address this issue, iPSC-derived MSCs have been developed as a step toward creating standardized off the-shelf therapeutic products.^28^ In the present study, we aimed to generate iPSC-derived induced chondrocyte progenitors (iCHOp) with a phenotype closely resembling primary ACI chondrocytes, offering a potentially suitable cell type for repair therapies for traumatic cartilage injuries. The A2B iPSC line, derived from human chondrocytes, has previously been characterized and shown to express pluripotency markers and the ability to differentiate into tissues representing the 3 different germ layers.^12^ This line was directed toward chondrocyte differentiation using 2 approaches: co-culture with irradiated chondrocytes^29^ and directed differentiation following the Oldershaw protocol.^12,14^

The A2B-iCHOp were characterized for their chondrogenic differentiation potential, and their transcriptomic profile was compared with that of undifferentiated human iPSC and surplus adult human chondrocytes isolated from the knee joints of patients undergoing ACI treatment.

Over the years, 3 main pathways for differenting iPSC or human embryonic stem cells into chondrocytes have been developed: via Embryoid body formation, MSCs, or mesodermal and Neural crest pathways. In this study, the focus was on developing a reproducable, xeno-free protocol suitable for bioreactor production. After thorough investigation of existing protocols, we excluded the embryoid body method and prioritized single cell protocols. Our results indicated that a combination of mesodermal factor Wnt3a, Neural crest-associated SMAD inhibitors and FGF10, each previousy shown to promote chondrogenesis, produced the best outcomes. The protocol aimed to partially replicate early fetal cartilage development by initially guiding iPSC toward mesenchymal/mesodermal cell lineages.

We based our approach on limb bud formation during embryogenesis, where cartilage precursors form a chondrogenic core while connective tissues differentiate peripherally.^30-33^ The limb bud ectoderm secretes Wnt3a and FGFs, which were incorporated into our protocol to promote proliferation while maintaining multipotency by downregulating SOX9, a key regulator of cartilage development.^34^ Our gene expression data suggested epithelial-to-mesenchymal transition, marked by Snai2 upregulation and shifts in cadherin expression, while SOX9 upregulation in iCHOp 3D cultures confirmed chondrocyte differentiation. SOX9 is essential for lineage commitment, survival, and activating cartilage-specific ECM genes such as aggrecan and collagen.^35,36^ The protocol also increased versican expression to levels comparable to ACI chondrocytes. Versican and other ECM molecules such as tenascin, syndecans, and N-CAM facilitate mesenchymal condensation and chondrocyte lineage commitment, aided by signaling molecules such as TGFβ, BMPs, and GDF5.^37-40^ During this process, synovial joints form as mesenchymal cells organize into interzones where the cells remain undifferentiated.^41,42^

Short-term Wnt/FGF signaling (<4 days) supports chondrogenic potential, whereas prolonged exposure redirects cells toward soft connective tissue. Our protocol mimicked early limb bud expression, incorporating dorsomorphin, a BMP pathway inhibitor, to balance Wnt and BMP effects on SOX9.^31,43^ To characterize the cells, we used markers like N-cadherin, Snai2, and PDGFRB, a mesenchymal cell marker that promotes chondrocyte proliferation and proteoglycan production without affecting collagen synthesis.^44^ This combination optimized the differentiation protocol, replicating conditions for effective chondrogenic development.

RNA sequencing, RT-qPCR, and GSEA demonstrated the iCHOp’s strong capacity for chondrogenesis, with over 83% gene expression similarity to human ACI chondrocytes. The analysis confirmed the activation of cartilage-specific pathways, including GDF5, which plays a key role in joint formation, chondrogenic differentiation, and cartilage repair, while mitigating OA-related degeneration.^45^ Expression of SOX9 and GDF5 in iCHOp after 3D culture highlights the protocol’s potential for generating functional chondrocytes for *in vivo* applications. Histological analysis further validated ECM production, including proteoglycans and collagen, confirming successful maturation.

The high versican/aggrecan ratio in iCHOp-derived matrix may enhance early cartilage regeneration by promoting cell migration, tissue sculpting, and integration with native cartilage, offering promise for therapeutic applications.^46^

A key outcome of this study is the demonstrated reproducibility of the protocol, as iCHOp derived from 3 distinct iPSC lines were successfully expanded in both serum-containing medium and a defined serum-free medium (PurStem 2) following 14 days of differentiation. In addition, the derivation protocol was successfully transferred to another research site, the University of Galway, where it yielded comparable results in chondrogenesis. This provides a solid foundation for further optimization and the potential use of iCHOp in future clinical applications.

## Conclusion

In conclusion, this study presents an optimized protocol for differentiation of iPSC into iCHOp, evaluated by transcriptomic profiling and histological characterization. Gene analysis confirmed successful differentiation toward a cartilage lineage, marked by increased expression of cartilage-specific ECM components during 3D culture, although the iCHOp exhibited a fetal chondrocyte phenotype characterized by versican expression. A significant contribution of this study is the demonstrated reproducibility of iCHOp derivation and culture in serum-free medium across 2 additional iPSC lines and reproducibility of results in another research site. We hypothesize that iCHOp with a more naïve phenotype may offer enhanced benefits for future cell therapies for local traumatic cartilage injuries, particularly by supporting a higher proliferative capacity and greater therapeutic potential. Furthermore, with increased regeneration potential the local cell repair may have a joint preservative effect and reduce the risk of further development of a localized traumatic injury to a secondary OA.

## Supplemental Material

sj-docx-1-car-10.1177_19476035251351713 – Supplemental material for Differentiation of Human Induced Pluripotent Stem Cells Toward Implantable Chondroprogenitor Cells Supplemental material, sj-docx-1-car-10.1177_19476035251351713 for Differentiation of Human Induced Pluripotent Stem Cells Toward Implantable Chondroprogenitor Cells by Josefine Ekholm, Kristina Vukusic, Camilla Brantsing, Georgina Shaw, Fazal Ur Rehman Bhatti, Stina Simonsson, Anna Falk, Mary Murphy, Victoria Rotter Sopasakis and Anders Lindahl in CARTILAGE

sj-docx-2-car-10.1177_19476035251351713 – Supplemental material for Differentiation of Human Induced Pluripotent Stem Cells Toward Implantable Chondroprogenitor Cells Supplemental material, sj-docx-2-car-10.1177_19476035251351713 for Differentiation of Human Induced Pluripotent Stem Cells Toward Implantable Chondroprogenitor Cells by Josefine Ekholm, Kristina Vukusic, Camilla Brantsing, Georgina Shaw, Fazal Ur Rehman Bhatti, Stina Simonsson, Anna Falk, Mary Murphy, Victoria Rotter Sopasakis and Anders Lindahl in CARTILAGE

sj-jpg-4-car-10.1177_19476035251351713 – Supplemental material for Differentiation of Human Induced Pluripotent Stem Cells Toward Implantable Chondroprogenitor Cells Supplemental material, sj-jpg-4-car-10.1177_19476035251351713 for Differentiation of Human Induced Pluripotent Stem Cells Toward Implantable Chondroprogenitor Cells by Josefine Ekholm, Kristina Vukusic, Camilla Brantsing, Georgina Shaw, Fazal Ur Rehman Bhatti, Stina Simonsson, Anna Falk, Mary Murphy, Victoria Rotter Sopasakis and Anders Lindahl in CARTILAGE

sj-xlsx-3-car-10.1177_19476035251351713 – Supplemental material for Differentiation of Human Induced Pluripotent Stem Cells Toward Implantable Chondroprogenitor Cells Supplemental material, sj-xlsx-3-car-10.1177_19476035251351713 for Differentiation of Human Induced Pluripotent Stem Cells Toward Implantable Chondroprogenitor Cells by Josefine Ekholm, Kristina Vukusic, Camilla Brantsing, Georgina Shaw, Fazal Ur Rehman Bhatti, Stina Simonsson, Anna Falk, Mary Murphy, Victoria Rotter Sopasakis and Anders Lindahl in CARTILAGE
