# Supplementary material for: Differentiation of Human Induced Pluripotent Stem Cells Toward Implantable Chondroprogenitor Cells
Source: Cartilage. 2025 Jul 3;17(3):644–59. doi: 10.1177/19476035251351713 (PMC12226525; doi:10.1177/19476035251351713)
Supplement: Supplementary file 1 [file sj-docx-1-car-10.1177_19476035251351713.docx]

**Supplementary table 1.** Antibodies used for Immunohistochemistry and Immunocytochemistry

| **Biomarker** | **Primary antibody** | **Clone** | **Manufacturer** | **Catalogue #** | **Dilution** |
| --- | --- | --- | --- | --- | --- |
| Nanog | Rabbit/IgG | D73G4 | Cell Signaling | 4903 | 1:800 |
| Oct 4A | Rabbit/IgG | C30A3 | Cell Signaling | 2840 | 1:400 |
| Versican | Rabbit/IgG | - | Boster Biological^1^ | PA1755 | 1:50 |
| Collagen III | Mouse/IgG1 | 1E7-D7/Col3 | Abcam | ab23445 | 1:100 |
| N-Cadherin | Mouse/IgG1 | 32 | BD Biosciences | 610920 | 1:50 |
| α Smooth Muscle Actin | Mouse/IgG2a | αSMA | Merck | CBL171 | 1:200 |
| Aggrecan | Mouse/IgG1 | - | ThermoFisher | AHP0022 | 1:250 |
|  |  |  |  |  |  |
|  |  |  |  |  |  |

Supplementary table 1 summarizes the information for the primary antibodies used for immunohisto- and immunocytochemistry. ^1^Boster Biological Technology.
