# Supplementary material for: Differentiation of Human Induced Pluripotent Stem Cells Toward Implantable Chondroprogenitor Cells
Source: Cartilage. 2025 Jul 3:19476035251351713. Online ahead of print. doi: 10.1177/19476035251351713 (PMC12226525; doi:10.1177/19476035251351713)
Supplement: sj-docx-2-car-10.1177_19476035251351713 – Supplemental material for Differentiation of Human Induced Pluripotent Stem Cells Toward Implantable Chondroprogenitor Cells [file sj-docx-2-car-10.1177_19476035251351713.docx]

**Supplementary table 2. TaqMan Gene Expression Assays**

| **Gene name** | **Gene symbol** | **Assay ID** | **Referred to in Fig. 3** |
| --- | --- | --- | --- |
| Aggrecan | ACAN | Hs00153936_m1 | Aggrecan |
| Cadherin2/N-cadherin | CDH2 | Hs00983056_m1 | CDH2 |
| E-cadherin 1 | CDH1 | Hs01023895_m1 | E-cadherin |
| Collagen type IIα1 | COL2A1 | Hs00156568_m1 | Collagen type 2A |
| Collagen type IIIα1 | COL3A1 | Hs00943809_m1 | Collagen type 3A |
| Growth differentiation factor-5 | GDF5 | Hs00167060_m1 | GDF5 |
| POU class 5 homeobox 1 pseudogene 3 | OCT4-pg3 /POU5F1 | Hs01895061_u1 | Oct4 |
| Platelet derived growth factor receptorβ | PDGFRB | Hs01019589_m1 | PDGFRB |
| Snai family transcriptional repressor 2 | SNAI2 | Hs00950344_m1 | SNAI2 |
| SRY-box 6 | SOX6 | Hs00264525_m1 | Sox6 |
| SRY-box 9 | SOX9 | Hs00165814_m1 | Sox9 |
| Versican | VCAN | Hs00171642_m1 | VCAN |
| Cyclophilin A | PPIA/CYPA | Hs99999904_m1 | CYPA |
|  |  |  |  |

Supplementary table 2 summarizes the Taqman gene expression assays, all from Applied Biosystems.
