# Supplementary figures and images for: Differentiation of Human Induced Pluripotent Stem Cells Toward Implantable Chondroprogenitor Cells
Source: Cartilage. 2025 Jul 3:19476035251351713. Online ahead of print. doi: 10.1177/19476035251351713 (PMC12226525; doi:10.1177/19476035251351713)

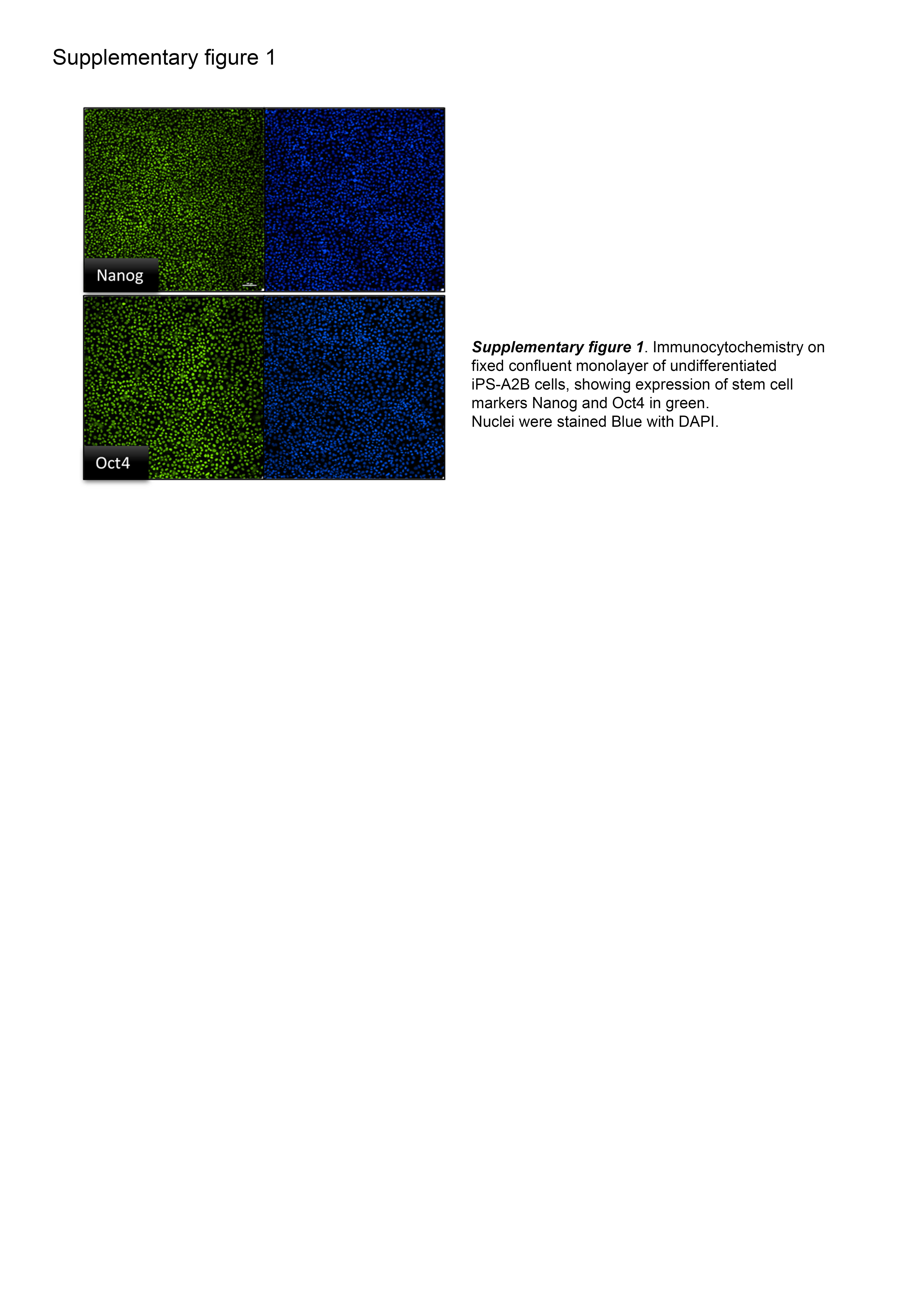

Supplement: sj-jpg-4-car-10.1177_19476035251351713 – Supplemental material for Differentiation of Human Induced Pluripotent Stem Cells Toward Implantable Chondroprogenitor Cells [file sj-jpg-4-car-10.1177_19476035251351713.jpg]
